# Zn nutrients-loaded chitosan nanocomposites and their efficacy as nanopriming agents for maize (*Zea mays*) seeds

**DOI:** 10.3389/fchem.2023.1243884

**Published:** 2023-08-11

**Authors:** Bongiwe Zungu, Hugues Kamdem Paumo, Joseph Lesibe Gaorongwe, Gaborone Neo Tsuene, Oziniel Ruzvidzo, Lebogang Katata-Seru

**Affiliations:** ^1^ Department of Chemistry, Material Science Innovation and Modelling (MaSIM) Research Focus Area, North-West University, Mmabatho, South Africa; ^2^ Department of Botany, School of Biological Sciences, North-West University, Potchefstroom, South Africa

**Keywords:** chitosan, nanocomposites, nanofertilizers, zinc oxide, nanopriming, germination traits, early seedlings development

## Abstract

Recent breakthroughs in agro-inputs research have led to the development of nanomaterials that can promote precision agriculture and better environmental security. The agricultural sector is increasingly facing the negative impacts of changing climates due to various stress conditions. To curb this scenario, economical and low-risk practices such as decreasing fertilizer inputs and seed priming have been promoted. In the current study, the *H. odoratissimum* aqueous extract was used to nucleate the Zn ionic species and grow the zinc oxide nanoparticles (ZnO NPs). The developed nanocomposites and their ionic zinc precursor were then integrated into tripolyphosphate (TPP)-crosslinked chitosan (CS/TPP) nanostructures by ionic gelation. Advanced physicochemical characterization techniques (SEM, EDS, TEM, DLS, FTIR, TGA, and XPS) were exploited to report the morphology, hydrodynamic size, surface charge, and structural organization of the developed nanomaterials. These revealed positively charged particles with hydrodynamic size in the 149–257 nm range. The NPs were used as priming agents for *Zea mays* seeds. At 0.04%, the ZnO-loaded CS/TPP NPs achieved higher root and shoot elongation in 10-day old seedlings compared to other treatments. The pristine CS/TPP NPs, Zn(II)-laden CS/TPP NPs, and ZnO-loaded CS/TPP NPs at 0.01% significantly promoted the early seedling development of seeds under salt stress. This represents the first report showing ZnO integrated chitosan nanocomposites as an auspicious nanopriming agent for stimulating the seed germination of maize. The study envisages offering perspectives on utilizing green nanotechnology to improve the early seedling development of maize. Furthermore, it has the potential to contribute towards UN SDG 2, thus addressing the threats to global food insecurity and doubling agricultural productivity by 2030.

## Graphical Abstract

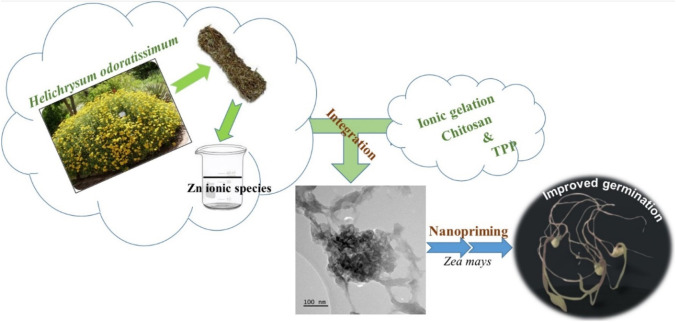

## 1 Introduction

Biopolymers are gaining close attention for the development of eco-friendly and sustainable products due to their innocuous nature, biodegradability, and large volume availability of the bioprecursors (Oladzadabbasabadi et al., 2022). Among these, chitosan represents one of the most abundant materials with versatile applications, including food packaging, beverages, and agriculture (MorinCrini et al., 2019). Chitosan is obtained from the structural modification (*N*-deacetylation) of chitin, a constituent of the extracellular matrix of arthropods, sponges, and fungi. Its molecular structure consists of *β*-(1→4)-2-acetamido-*D*-glucose and *β*-(1→4)-2-amino-*D*-glucose repeating units. The presence of hydroxyl and amino groups along the polymeric chain of chitosan influences its solubility and uses. The hydrogen bonding network of chitosan material is responsible for its low solubility in water, which is one of the major limitations in agricultural applications (Li et al., 2019). In an acidic milieu, the amino groups get protonated, and the prompted cationic matrix can interact with crosslinking agents to generate chitosan nanoscale particles with high sorption capability toward the water. Moreover, this process can also be exploited to encapsulate the potentially toxic nutrients plants need and achieve nanofertilizers with slow-release conduct.

Slow-release nanofertilizer is a concept recently adopted in the literature and by corporations to promote sustainable agriculture (Kubavat et al., 2020; Kumaraswamy et al., 2021). Indeed, conventional water-soluble inputs have been overly reported to exhibit low efficiency in plant nutrient absorption. Generally, they are associated with environmental burdens and health concerns. Nanofertilizers are designed to supply nutrients lethargically under controlling factors like hydrolysis and diffusion (Guo et al., 2018; Lawrencia et al., 2021). This technological innovation includes integrating the nutrients within nanoscale modules. For example, the commercially available nano-oxides CuO (30–50 nm) (Leonardi et al., 2021) and ZnO (10–30 nm) (Asgari-Targhi et al., 2021) have previously been reported for sustained release of Cu and Zn micronutrients, following their encapsulation within chitosan-alginate and chitosan nanostructures, respectively.

The conventional techniques of supplying nutrients, i.e., foliar spraying and soil fertilization, rely on environmental conditions that can lessen their efficiency. High soil pH, for example, limits the availability of micronutrients. Low moisture, on the other hand, may restrict their uptake by plants. In the case of the foliar-sprayed method, there is a high risk of phytotoxicity as multiple treatments are often required. To restrain these shortcomings, seed pretreatment (priming) with zinc-rich nanofertilizers has been described as an eco-efficient strategy that can enhance seedling establishment (Majda et al., 2019; Babu et al., 2022). Zn plays a major role in plants’ healthy development due to its participation in several enzymatic reactions and metabolic processes. This micronutrient acts as a regulatory co-factor of many enzymes, including oxidoreductases, hydrolases, ligases, transferases, and isomerases (Prasad et al., 2012).

The seed priming technique has shown commendable potential in crop production by improving the seed quality, which is essential for better stand establishment and higher yield, even under stress conditions (Rai-Kalal and Jajoo, 2021). For example, *Zea mays* seeds primed using chitosan solutions have demonstrated increased shoot height and dry weight under cold stress (Guan et al., 2009). Moreover, priming seeds of this cereal grain with Cu^2+^-encapsulated chitosan nanoparticles has been found to enhance the development of seedlings as well as the enzymatic antioxidant response under higher temperatures and relative humidity (Gomes et al., 2021). Under salt stress, priming lupine seeds with ZnO NPs (21 nm; concentrations 20, 40, and 60 mg/L in distilled water) increased the root length, shoot length, and fresh weight in 20-day old seedlings (Abdel Latef et al., 2017). Higher germination and increased radical length have been described in the salinized bean seeds primed with TPP-crosslinked CS NPs (46 nm; concentrations 0.1%, 0.2% and 0.3%) (Zayed et al., 2017). Studies have demonstrated that salt conditions inhibit seed germination, cause a nutritional imbalance in plants, and persuade the excessive production of ROS, which can degrade proteins and DNA structures (Manohara et al., 2022; He et al., 2018). A high concentration of NaCl in the soil is one of the most severe environmental factors that occur due to extensive irrigation with saline water and/or the extensive application of fertilizers. The development of low-cost, simple, and environmentally sound nanomaterials to prime seeds would render physiologically elegant seed treatments more practicable.

This study reports the development of ZnO-loaded CS and Z (II)-laden CS nanostructures as priming agents to improve the initial growth of non-stressed and salt stressed maize seedlings. A green process using the aqueous extract of *Helichrysum odoratissimum* L. in the presence of Zn(II) ions afforded ZnO NPs. Their integration into the CS/TPP NPs was performed through the ionic gelation method. Similarly, zinc nitrate solutions were also nano-formulated. The as-prepared Zn-containing nanomaterials were characterized by scanning electron microscopy (SEM), and energy dispersive X-ray spectroscopy (EDX). X-ray diffraction (XRD), Fourier transform infrared spectroscopy (FTIR), dynamic light scattering (DLS), and X-ray photoelectron spectroscopy (XPS) techniques. Their Zn release profile was examined in water by inductively coupled plasma-mass spectrometry (ICP-MS). A systematic evaluation of their effects as nanopriming agents to ameliorate *Zea mays* seed germination and seedling establishment was carried out.

## 2 Materials and methods

### 2.1 Materials

The aerial part of *Helichrysum odoratissimum* L. was acquired from a local market (Rustenburg, North-West, South Africa) and identified in the Department of Crop Science, North-West-University, South Africa. Zinc nitrate hexahydrate (Zn(NO_3_)_2_.6H_2_O) salt, chitosan powder (low molecular weight), sodium tripolyphosphate, and sodium hydroxide of high grade of purity were all purchased from Sigma-Aldrich (St. Louis, MO, USA). All aqueous solutions were prepared using deionized water.

### 2.2 Synthesis of ZnO nanoparticles

50 mL of an aqueous solution of Zn(NO_3_)_2_.6H_2_O (3.3 mmol L^−1^) was added dropwise into 100 mL of a stirred (300 rpm) solution of *H. odoratissimum* extract, prepared by heating 10 g of dried plant material in deionized water at 70°C for 1 h. The mixture was allowed to react at 50°C for 24 h, and the pH of the obtained suspension was adjusted to 10 using a solution of NaOH (1 mol L^−1^). The resultant colloid was centrifuged at 6 000 rpm and washed thrice using deionized water. The precipitate obtained was dried at 80°C for 24 h to generate a material designated as ZnO [10s]. This procedure was repeated using 15 g of plant material, and the product isolated was named ZnO [15s].

### 2.3 Integration of Zn nutrient within TPP-crosslinked CS nanoparticles

ZnO [10s] and ZnO [15s] were treated with CS solution in the presence of TPP dissolved in water. In a typical procedure, 0.30 g of CS powder was dissolved in 25 mL of acetic acid (1%, v/v) under magnetic stirring at room temperature. Subsequently, 0.10 g of ZnO [10s] was slowly added, and the mixture was treated with 5 mL of TPP (1%, w/v) dropwise under continuous stirring (300 rpm) for 30 min. Thereafter, the opalescent solution was centrifuged at 6 000 rpm, and the precipitate obtained was freeze-dried to generate ZnO [10s]-loaded CS/TPP. A similar procedure was followed using ZnO [15s], which afforded ZnO [15s]-loaded CS/TPP. 0.10 g of Zn(NO_3_)_2_.6H_2_O was also treated as described for the synthesis of ZnO [10s]-loaded CS/TPP to afford Zn(II)-laden CS/TPP. Considering that Zn nutrient was introduced as ionic species in this case, the complexation efficiency (CE) was determined as follows:

$$CE\;\left(\%\right)=\frac{TZn-FZn}{TZn}\times 100$$

where, TZn and FZn denote the total amount of Zn used during the preparation of the Zn(II)-laden CS/TPP NPs and the fraction of free Zn in the supernatant after centrifugation. FZn was obtained *via* an inductively coupled plasma-mass spectrometer (ICP-MS) equipment (Agilent 7,500 series spectrometer). The pristine CS/TPP NPs were also prepared upon mixing the biopolymer solution and TPP crosslinker.

### 2.4 Characterization techniques

The as-prepared nanoparticles were characterized by X-ray diffraction (Bruker D8 Advance with Cu Kα radiation (*λ* = 0.154 nm)), SEM imaging (Zeiss Ultra Plus FE-SEM), TEM investigation (JEOL JEM 2100 at 200 kV), and EDX analysis (AZtecEnergy software integrated to an Oxford detector). The particle size, polydispersity index (PDI), and zeta potential (ZP) were determined by DLS measurements (Malvern Nano ZS-90). The compositional and structural modification was described using FTIR (Bruker Alpha) and XPS (Thermo Fisher Scientific spectrometer ESCAlab 250Xi with a monochromatic Al kα (1486,7 eV) X-ray source) spectra interpretation. Thermogravimetric-Differential Scanning Calorimetry (Simultaneous TGA/DSC SDT-Q600) was performed at a 10°C/min ramp rate in N_2_ environment.

The Zn-release behaviour of the prepared nanomaterials was also investigated using the teabag technique (Ekanayake and Godakumbura, 2021). The ZnO [15s], ZnO [15s]-loaded CS/TPP, and Zn(II)-laden CS/TPP were chosen as specimens. 30 mg of each sample was lodged into an empty teabag, placed in a beaker containing 50 mL of tap water (pH 7.8-8.1), and then allowed to settle. The variation in the Zn concentration was assessed for 02 weeks, and each experiment was carried out thrice. After 01, 05, 24, 48, 168, 240, and 336 h, aliquots (10 mL) were collected using the ICP-MS technique for Zn analysis. Each time an aliquot was removed, deionized water of the same volume was added to the beaker to keep the volume constant.

### 2.5 Protocol for seed priming

The *Zea mays* seeds of R450w/uo2250w CML550 variety were provided by Molelwane Farm, Department of Crop Science, North-West University, South Africa. 81 seeds were then selected, cleaned and surface-sterilized using 70% (v/v) ethanol and 1.25% (v/v) sodium hypochlorite solution. Thereafter, these seeds were rinsed three times with distilled water. Next, the sterilized seeds were kept in deionized water at 4°C for 48 h to achieve stratification. For priming solutions, appropriate amounts of ZnO [15s], CS/TPP, ZnO [10s]-loaded CS/TPP, ZnO [15s]-loaded CS/TPP, and Zn(II)-laden CS/TPP NPs were weighed to prepare 0.01% and 0.04% concentrations in deionized water. Duplicates were also prepared in 600 mM NaCl aqueous solution. Seeds (09) were then imbibed in different concentrations at 25°C for 4 h. Seeds primed with deionized water were used as the control.

### 2.6 Germination study

The germination tests were conducted in triplicates for each treatment group, and a series of 03 seeds from each solution was placed onto a moistened sterile Whatman filter paper in a transparent petri dish (90 × 15 mm). All prepared petri dishes were then arranged in a TL-300 Growth Chamber (Jeio Tech, Seoul, Korea) at 23°C. The relative humidity was maintained at 70% under 150–650 μmol s^−1^m^−2^ artificial light. The ambient CO_2_ was set to range between 380–390 ppm. Periodic wetting of the filter papers was achieved using 2.0 mL of deionized water. After 10 days of incubation, the following growth traits were evaluated: coleoptile length (CL), length of roots (LoR), number of lateral roots (NLR) on radicle, number of roots (NR) additional to the radicle, radicle length (RL), shoot length (SL), coleoptile dry weight (CDW), radicle dry weight (RDW), shoot dry weight (SDW), and root dry weight (RoDW).

## 3 Results and discussion

The structural characteristic of chitosan makes it suitable for metal ions binding through complexation (Deshpande et al., 2017). The small radius-to-charge ratio of Zn(II) ions is accountable for their pronounced Lewis acid characteristic, which justifies their ability to stabilize with N- and O-donors. All studies on the plant extract-mediated synthesis of metal and metal oxide NPs are often associated with the presence of numerous biomolecules such as amino acids, phenolic acids, alkaloids, terpenoids, alcoholic compounds, polysaccharides, and various chelating proteins (Jeevanandam et al., 2016; Pradeep et al., 2022), which are collectively predicted to participate in the reduction of metal ions into nanoparticles.

In the present study, nanostructured chitosan may act to control the Zn release and improve their assimilation by plants (Jimenez-Rosado et al., 2022). Moreover, chitosan has been reported to significantly enhance the seedling growth of several plants, including wheat (*Triticum aestivum* L.) (Wang et al., 2015), rice (*Oryza sativa* L.) (Chamnanmanoontham et al., 2015; Divya et al., 2022), and maize (*Zea mays* L.) (Agbodjato et al., 2016). In a recent study published by our team (Dhlamini et al., 2020), soil amendment using CS/TPP NPs resulted in increased plant height of maize, 10 weeks after planting. Therefore, the combinative Zn micronutrient-CS/TPP NPs may enhance the plants’ physiological parameters even better.

**FIGURE 1:**
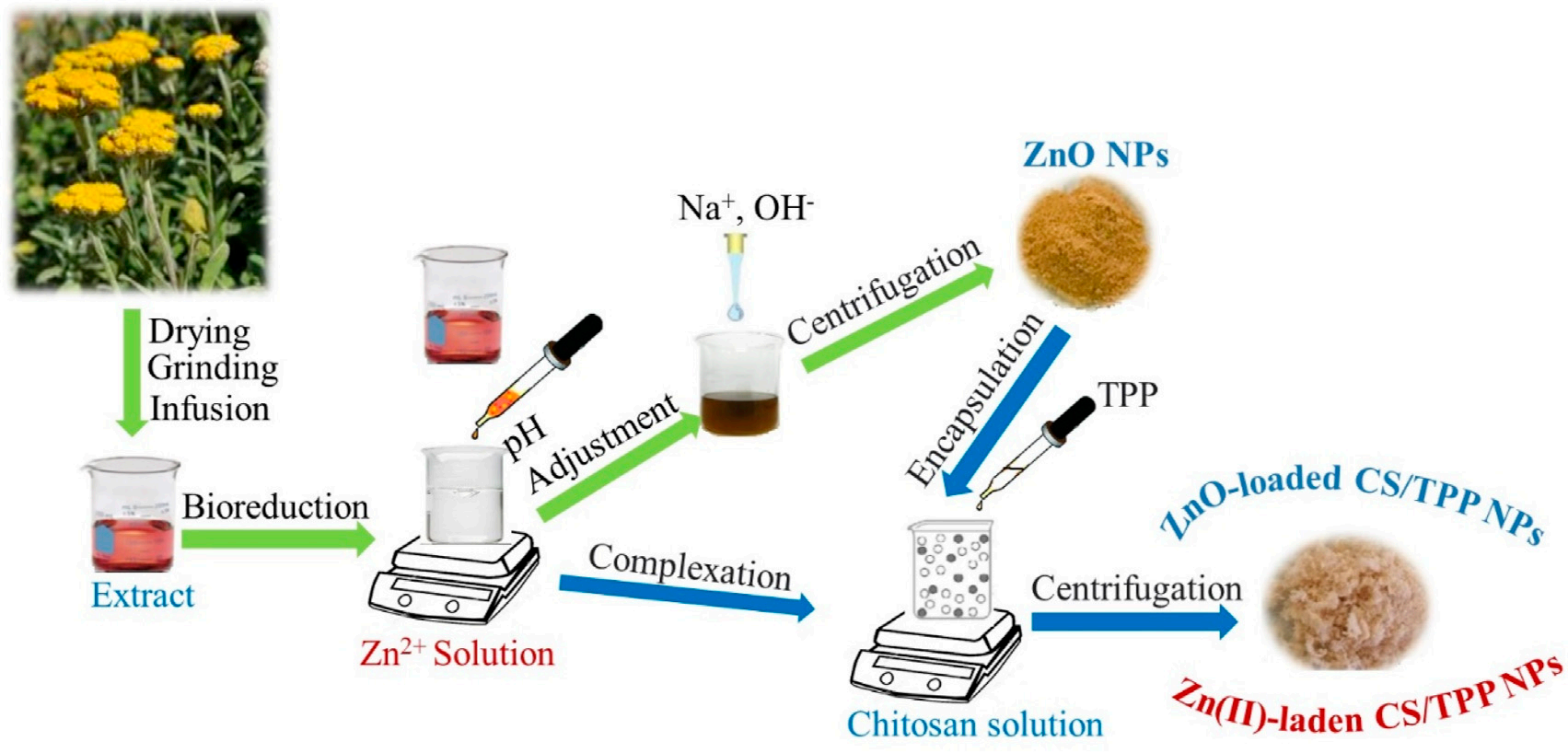
Schematic illustration for the preparation of Zn-loaded chitosan nanostructures.

*H. odoratissimum* is an aromatic shrub belonging to the Asteraceae’s family and native to the southern region of Africa. This plant is frequently used as incense during cultural rites. The health benefits of *H. odoratissimum* are also well-reported, including their antifungal, antioxidant, hepatoprotection, hypoglycemic, and cytotoxicity activities (Lourens et al., 2008; Akinfenwa et al., 2022). These therapeutic properties suggest that the extract of *H. odoratissimum* contains phytochemicals that could form complexes with Zn ionic species and promote their conversion to Zn-containing nanoparticles (Abomuti et al., 2021). The aqueous extracts of *H. odoratissimum* could also act as a reducer and oxygen provider for the nucleation and growth of nano-zinc oxides during the aging operation. The latest sustainability initiatives encourage the use of environment-friendly procedures for the synthesis of nanoparticles. The steps carried out to fabricate Zn-loaded chitosan nanoparticles are depicted in Figure 1.

### 3.1 Characterization of the developed nanomaterials

DLS analysis revealed ZnO NPs with average hydrodynamic sizes of 173 and 144 nm for samples obtained using 10 g (ZnO [10s]) and 15 g (ZnO [15s]) of plant materials, respectively (Table 1). As the concentration of phytochemicals increases with the amount of *H. odoratissimum*, the size of ZnO [15s] were decreased. This is presumably attributed to the high degree of complexation and stabilizing effect of phytochemicals towards Zn(II) ions at a relatively high concentration. Treatment of these nano-oxides and their precursor (Zn^2+^, NO_3_^−^) in acidic chitosan solution in the presence of TPP crosslinking agent generated particles with larger sizes 195–257 nm. The hydrodynamic size of the CS/TPP NPs was significantly increased when Zn loading was achieved. The average uniformity of the free nano-oxide and oxide-loaded chitosan-based nanostructures in distilled water was also estimated, and the PDI ranges from 0.197 to 0.373. The oxide-loaded CS/TPP NPs and Zn(II)-laden CS/TPP NPs displayed values below 0.3. These are more often considered acceptable for polymeric nanomaterials (Danaei et al., 2018). The free ZnO NPs showed a negative surface charge, while the CS/TPP NPs, ZnO-loaded CS/TPP NPs, and Zn(II)-laden CS/TPP NPs exhibited a positive charge due to the presence of ionized amino groups of chitosan, which formed the shell of the CS/TPP NPs. These developed through the formation of CS-TPP complexes, which are further bridge crosslinked into ordered colloids by excess TPP. The ZP value of the ZnO [10s]- and ZnO [15s]-loaded CS/TPP NPs was lower than that of the bare ZnO NPs and pristine CS/TPP NPs due to the electrostatic interactions ZnO NPs(−)→ ←(+)CS/TPP NPs in the mixed system.

**TABLE 1:** Average hydrodynamic size, PDI, and ZP of colloidal suspensions of CS/TPP and nutrient-loaded CS/TPP NPs.

Nanomaterial	Size (nm)	PDI	ZP (mV)
ZnO [10s] NPs	173.5 ± 7.79	0.373	−26.6
ZnO [15s] NPs	144.6 ± 2.60	0.325	−34.2
ZnO [10s]-loaded CS/TPP NPs	257.3 ± 19.89	0.197	17.8
ZnO [15s]-loaded CS/TPP NPs	207.2 ± 18.53	0.226	16.0
Zn(II)-laden CS/TPP NPs	195.0 ± 6.425	0.221	21.5
CS/TPP NPs	149.4 ± 2.239	0.410	19.7

**FIGURE 2:**
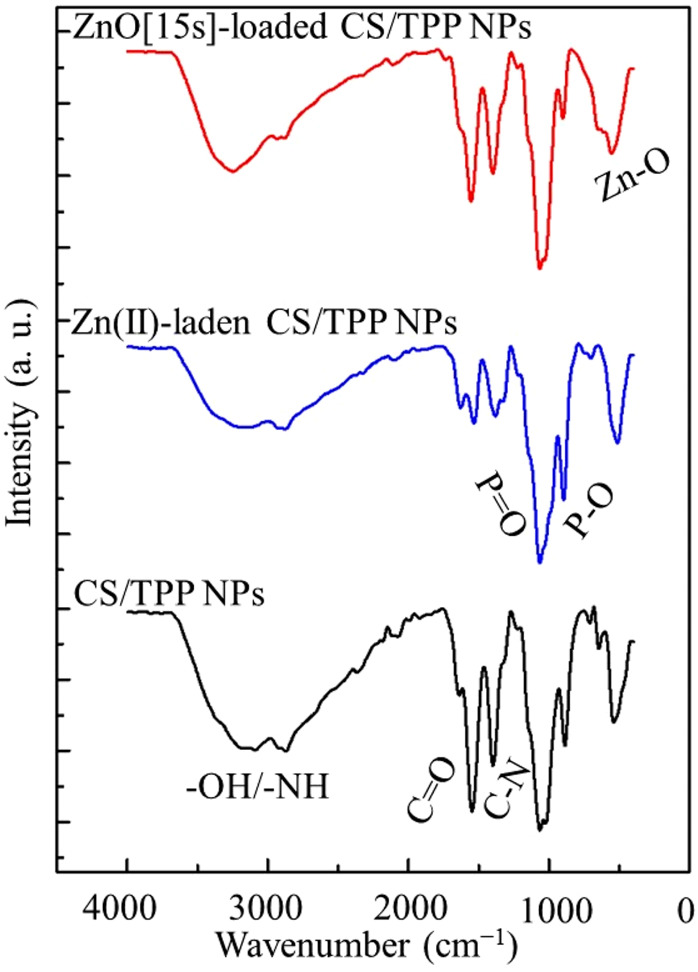
FTIR spectra of the CS/TPP, ZnO [15s]-loaded CS/TPP, and Zn(II)-laden CS/TPP NPs.

The FTIR spectrum of CS/TPP nanoparticles illustrated in Figure 2 displays broad absorption bands at 3,177 and 2,883 cm^−1^ due to the elongation vibrations of–OH and–NH bonds in the biopolymer matrix. The bands at 1,533 and 1,392 cm^−1^ are assigned to the C=O and C–N stretching modes, respectively. The bands detected at 1,052 and 1,008 cm^−1^ indicate the presence of TPP crosslinker (Rahmani et al., 2017; Aşık et al., 2021). These bands are attributed to the stretching vibration of the P=O and P–O bonds. The main differences observed between spectra of the CS/TPP NPs and the Zn(II)-laden CS/TPP NPs include a drop in intensity of the bands corresponding to the O–H/N–H and C=O/C–N stretching frequencies, reflecting the complexation of Zn(II) ions with the heteroatoms of these functional groups. As for the spectrum of ZnO [15s]-CS/TPP NPs, sharp bands observed at 479 cm^−1^ were ascribed to the stretching vibration of the Zn–O bond (Ndayishimiye et al., 2021). Furthermore, no significant difference was recorded between the FTIR spectra of ZnO [15s]-CS/TPP NPs and ZnO [10s]-CS/TPP NPs.

**FIGURE 3:**
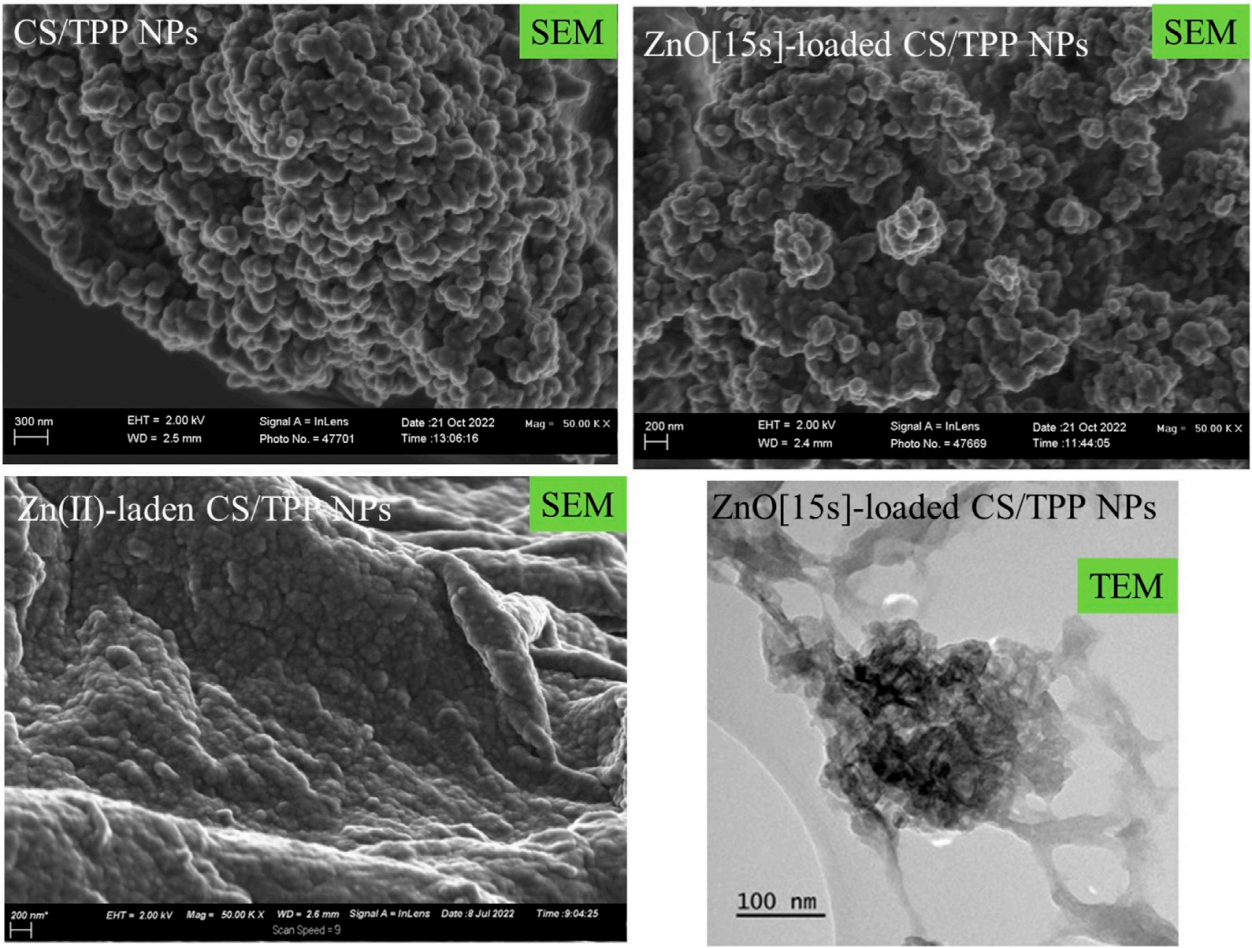
SEM images of the CS/TPP, ZnO [15s]-loaded CS/TPP, and Zn(II)-laden CS/TPP NPs; and TEM image of the ZnO [15s]-loaded CS/TPP NPs showing the encapsulation of ZnO.

**FIGURE 4:**
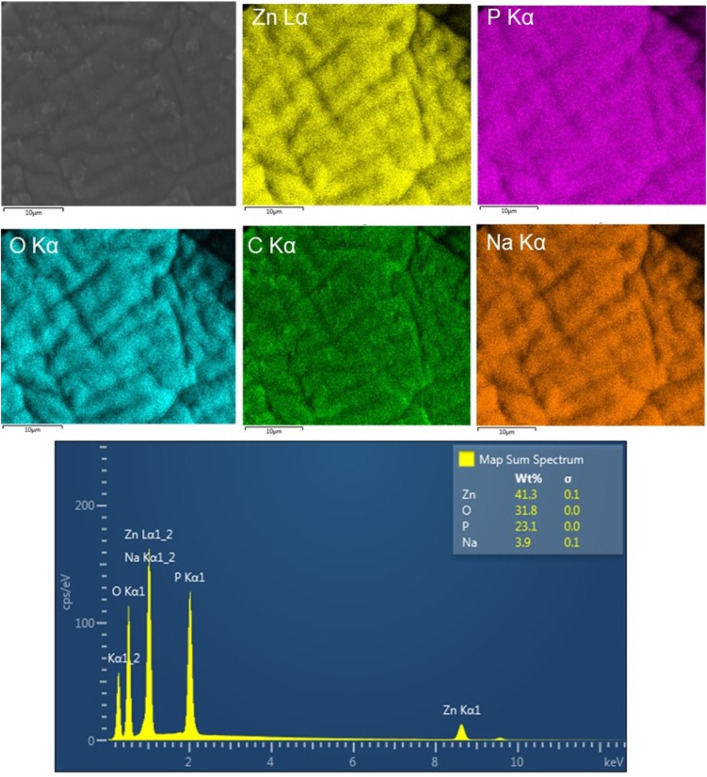
Elemental maps (Zn, P, O, C, and Na) and EDX spectrum of Zn(II)-laden CS/TPP NPs.

The morphological characteristic of these CS-based nanoformulations was also examined using SEM and TEM images (Figure 3). SEM image of the CS/TPP NPs reveals pseudo-spherical shapes of various sizes. SEM micrograph of the ZnO [15s]-loaded CS/TPP NPs, on the other hand, presents uneven nano-aggregate shapes, suggesting a higher degree of electrostatic attraction at some areas of the visualized sample. In addition, the TEM image of this nanocomposite establishes the efficacy of the ionic gelation procedure to induce the agglomeration of CS nanostructures during the introduction of ZnO nanopowders. This observation is in accordance with the potential measured for the ZnO NPs (negatively charged) and CS/TPP NPs (positively charged). Contrastingly, the SEM image of the Zn(II)-laden CS/TPP NPs shows spherical-like shapes in a superior gelling system. Zinc di-cation species undergo complexation with the amino and hydroxyl groups on the chitosan scaffold, increasing the viscosity of the polymeric solution and organization of the CS/TPP network (Xing et al., 2014). The complexation efficiency calculation shows that 46.1% of Zn was available in the integrated form within the CS/TPP NPs. EDX analysis of this sample shows peaks corresponding to Zn, P, O, and C elements at energy values of 1.012, 2.013, 0.525, and 0.277 keV, respectively (Figure 4). The corresponding elemental maps indicate a uniform distribution. For Zn, the measured weight% was higher by 41,3% on the surface.

**FIGURE 5:**
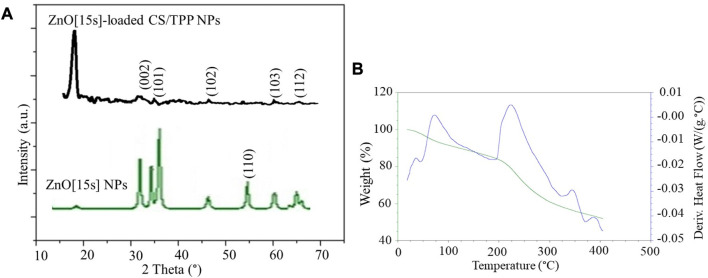
**(A)** XRD patterns of the ZnO [15s] and ZnO [15s]-loaded CS/TPP NPs; **(B)** TGA/DSC plots of ZnO [15s]-loaded CS/TPP NPs.

XRD patterns of the ZnO [15s] and ZnO [15s]-loaded CS/TPP NPs (Figure 5A) show peaks at 2θ 34.36°, 36.37°, 46.46°, 60.61°, and 66.06°, attributed to (002), (101), (102), (103), and (112) reflections of ZnO with hexagonal wurtzite structure (Padalia and Chanda, 2017). The intense peak at 2θ 19.79° is due to crystalline CS nanostructures (ElNaggar et al., 2022). TGA/DSC thermograms of the ZnO [15s]-loaded CS/TPP NPs are presented in Figure 5B. The thermal decomposition pattern observed is divided into several key stages. An initial mass loss of 12.6% between 25 and 180 °C is due to the loss of surface and absorbed water molecules (Chigondo et al., 2018). The hygroscopic nature of CS/TPP nanostructures is responsible for the ability of nutrient-loaded CS/TPP NPs to absorb moisture. The most apparent weight loss stage of 31.4% recorded around 200–300 °C is attributed to ether bond cleavage in the chitosan skeleton (Naushad et al., 2021). The weight loss (5.3%) over the temperature range of 300°C–400°C is most likely due to the cleavage of O–H and N–H bonds (Kahdestani et al., 2021). Above 400°C, no further change in mass occurred. The XRD and TGA indicate that the pre-synthesized zinc oxide nanopowder was integrated into the CS/TPP NPs.

**FIGURE 6:**
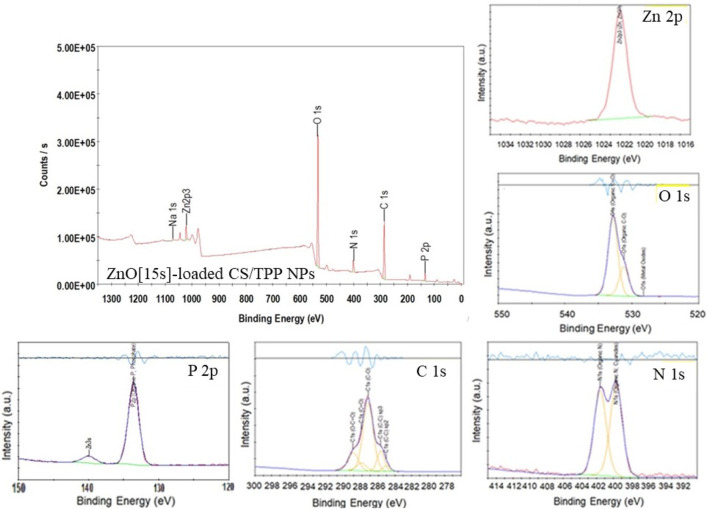
XPS survey spectrum of ZnO [15s]-loaded CS/TPP NPs; and high-resolution spectra of Zn 2p, C 1s, O 1 s, N 1s, and P 2p.

**FIGURE 7:**
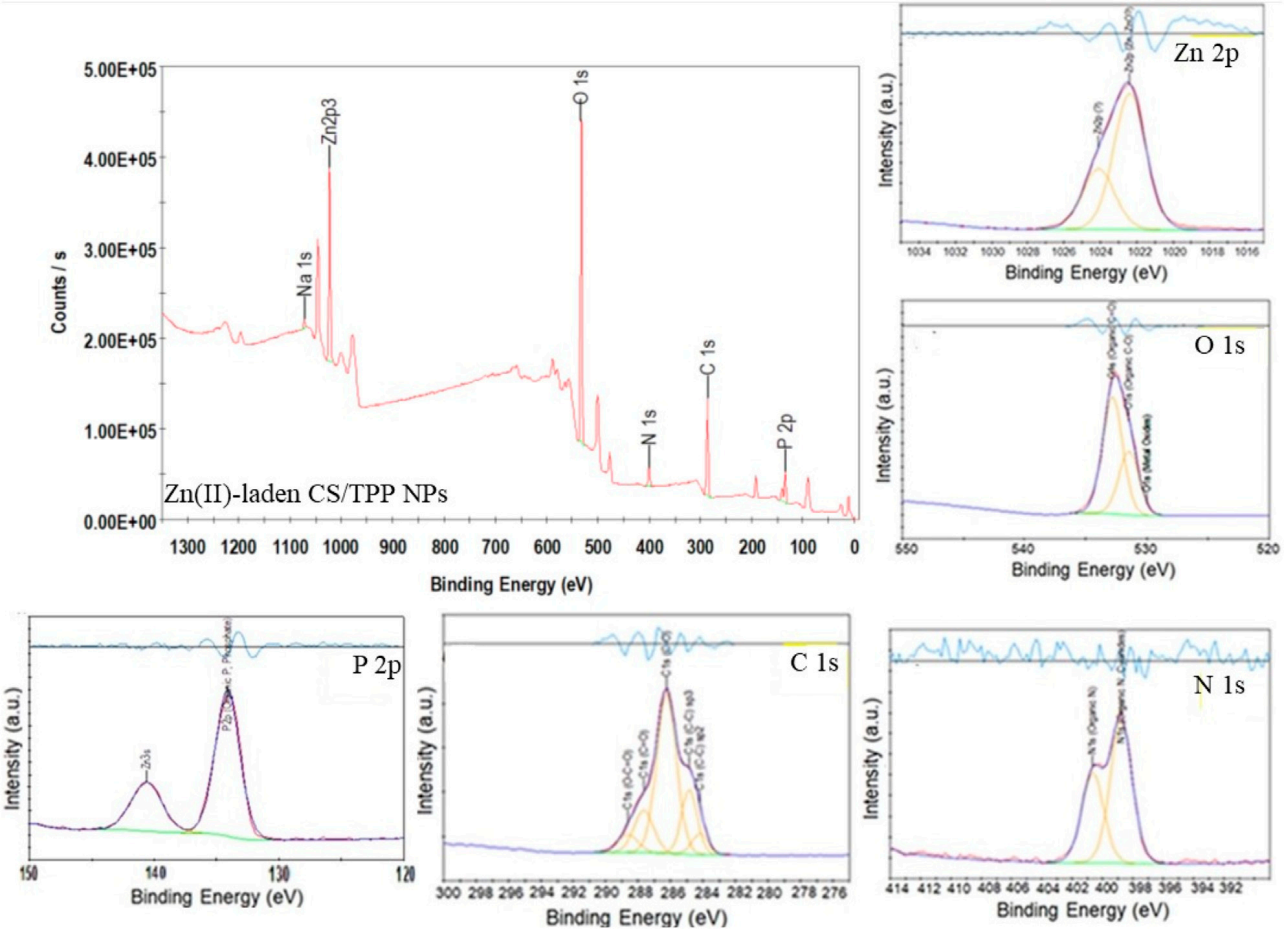
XPS survey spectrum of Zn(II)-laden CS/TPP NPs; and high-resolution spectra of Zn 2p, C 1s, O 1 s, N 1s, and P 2p.

XPS was used to confirm the composition of the nutrient-loaded CS/TPP NPs. The XPS survey spectra of the ZnO [15s]-loaded CS/TPP and Zn(II)-laden CSTPP NPs show the presence of Zn, O, C, P, N, and Na (Figures 6, 7). The presence of Na originates from an excess crosslinking agent even after washing the nanoproducts several times with deionized water. Figure 6 also displays the XPS spectra of ZnO-loaded CS/TPP NPs at the core-level C 1s, O 1s, N 1s, and Zn 2p peaks. The C 1s peak was resolved into C=C (284.1 eV), C–C (284.7 eV), C–O (286.3 eV), C=O (287.1 eV), and O–C=O (288.1 eV) bonds (Rathore et al., 2021). The N 1s spectrum exhibits two deconvoluted peaks at 399.7 and 401.6 eV, contributions of–NH_2_ and protonated–NH_3_^+^ amine groups (Frindy et al., 2015; Zaman et al., 2022). The high-resolution spectrum of O 1s was deconvoluted into three peaks at 528.3, 531.2, and 532.8 eV assigned to Zn–O, C–O, and C=O bonds, respectively (Daniyal et al., 2021). The peaks at 133.6 and 1,022.0 eV are contributions by P 2p of phosphate (Qiao et al., 2015) and Zn 2p^3/2^ of Zn^2+^ in the ZnO lattice, respectively (Abdullin et al., 2021).

Table 2 summarizes the location and assignment of characteristic peaks for the ZnO [15s]-loaded CS/TPP NPs. Similar parameters are recorded with Zn(II)-laden CS/TPP NPs except for data obtained from the curve fitting of O 1s and spectrum of Zn 2p. While no deconvoluted peak attributed to the Zn–O bond (O^2-^ ions of ZnO structure) is observed, the high-resolution Zn 2p spectrum of Zn(II)-laden CS/TPP NPs can be deconvoluted to two peaks at 1,022.4 and 1,024.1 eV, suggesting dative coordination bonds of Zn^2+^ to N- and O-containing functional groups (Frindy et al., 2015). The parameters obtained through the individual peak profile fittings are shown in Figure 7 and summarized in Table 3. The XPS results confirm that the combinative ZnO/CS NPs and Zn(II) ions/CS NPs have been developed successfully.

**TABLE 2:** Characteristics of the XPS peaks observed for ZnO [15s]-loaded CS/TPP NPs.

Signal	Binding energy (eV)	FWHM (eV)	Atomic %
P 2p (Phosphate)	133.6	1.7	4.4
C 1s (C=C)	284.1	1.0	1.7
C 1s (C–C)	284.7	1.0	6.0
C 1s (C–O)	286.3	1.4	29.2
C 1s (C=O)	287.1	1.4	3.3
C 1s (O–C=O)	288.1	1.4	7.6
N 1s (Organic N)	399.7	1.7	3.4
N 1s (Organic N)	401.6	1.7	3.2
O 1s (Zn–O)	528.3	1.7	0.1
O 1s (C–O)	531.2	1.7	11.1
O 1s (C=O)	532.8	1.7	28.8
Zn 2p3 (Zn–O)	1,022.0	1.6	0.8
Na 1s (Na TPP)	1,071.4	1.8	0.3

FWHM: full width at half maximum.

**TABLE 3:** Characteristics of the XPS peaks observed for Zn(II)-laden CS/TPP NPs.

Signal	Binding energy (eV)	FWHM (eV)	Atomic %
P 2p (Phosphate)	134.1	2.3	7.7
Zn 3s	140.6	2.9	5.5
C 1s (C=C)	284.2	1.1	2.2
C 1s (C–C)	284.8	1.1	6.5
C 1s (C–O)	286.3	1.4	19.9
C 1s (C=O)	287.7	1.4	5.3
C 1s (O–C=O)	288.6	1.4	2.2
N 1s (Organic N)	399.8	1.7	2.8
N 1s (Organic N)	401.6	1.7	1.8
O 1s (C–O)	531.5	1.9	14.8
O 1s (C=O)	532.8	1.9	27.4
Zn 2p (Zn^2+^ complex)	1,022.4	2.1	2.4
Zn 2p (Zn^2+^ complex)	1,024.1	2.1	1.1
Na 1s (Na TPP)	1,071.8	2.1	0.5

### 3.2 Zn release behaviour of the developed nanomaterials

**FIGURE 8:**
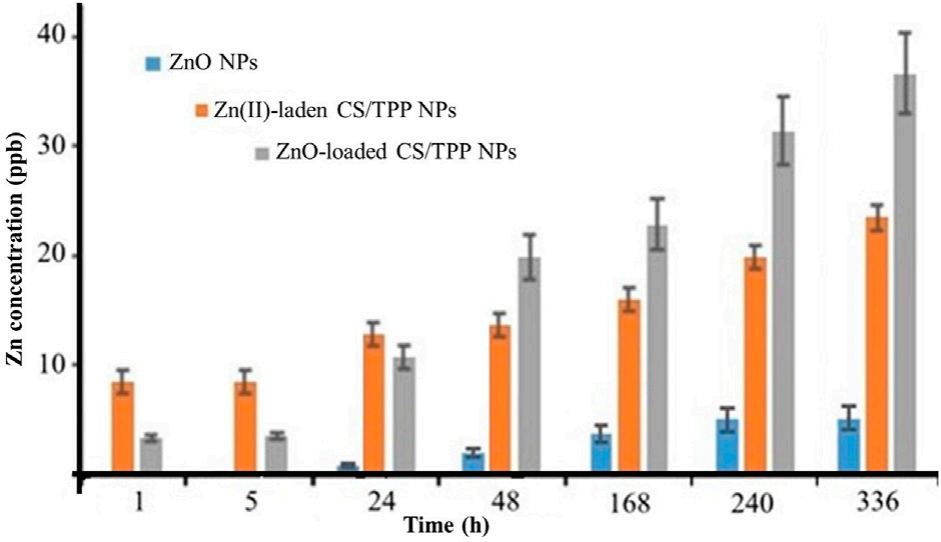
Plot depicting the Zn release behaviour of the ZnO [15s], ZnO [15s]-loaded CS/TPP, and Zn(II)-laden CS/TPP NPs in tap water.

Figure 8 illustrates the change in zinc concentrations for the prepared ZnO [15s], ZnO [15s]-loaded CS/TPP, and Zn(II)-laden CS/TPP NPs suspended in aqueous milieu for 02 weeks. Throughout this experiment, the amount of Zn(II) ions released was relatively low for the ZnO NPs compared to the ZnO [15s]-loaded CS/TPP and Zn(II)-laden CS/TPP NPs. This difference in behaviour is due to the reduced solubility of crystalline ZnO NPs in water at high pH. However, this phenomenon is modulated after chitosan encapsulation to generate amorphous nanostructures. The entrapment of ZnO NPs during crosslinking reaction makes it low in crystallinity (Matsumoto et al., 2002), as supported by the X-ray diffractogram of the ZnO [15s]-loaded CS/TPP NPs (Figure 5A). Relatively high concentrations of Zn(II) ions were observed for the Zn(II)-laden CS/TPP NPs after 01, 05, and 24 h of immersion. On the other hand, the 48, 168, 240, and 336 h release patterns showed higher Zn concentrations for the ZnO [15s]-loaded CS/TPP NPs. Furthermore, solutions containing this nanocomposite exhibited a gradual increment in Zn concentrations while their Zn(II)-laden counterparts showed a rapid release of Zn. Ekanayake and Godakumbura (2021) reported that metal oxide NPs integrated into the alginate matrix can be held by weaker interactions (hydrogen bonding). The gradual change in Zn concentration of the ZnO [15s]-loaded CS/TPP NPs in water is due to CS/TPP swelling, dissolution of the amorphous ZnO, and diffusion of Zn from the swollen matrix in analogy with the literature precedent for the alginate-polyvinyl alcohol loaded with ZnO NPs (Knijnenburg et al., 2021). The early burst release behavior of Zn(II)-laden CS/TPP NPs can be attributed to the diffusion of Zn at the surface of the particles. Earlier, evidence on the slow P release in soil from the 430 nm CS/TPP-ZnO nanocomposites was also reported by Dimkpa et al. (2023). These nanomaterials were developed by the ionic gelation method in the presence of the commercialy available 18 nm ZnO NPs.

### 3.3 Priming effects of the developed CS-based NPs on seed germination and seedling growth

The effects of micronutrient-based seed priming treatments on seed germination and post-germination seedling growth have been extensively investigated (Rizwan et al., 2017). Seed priming in a solution containing nanoparticles (nanopriming) is an innovative technique to accelerate and synchronize germination. Seed treatment under controlled conditions before sowing can also enhance plant growth and increase their resistance to abiotic stress conditions. In so doing, nanopriming can help reduce the overapplication of fertilizers. For example, nanopriming of *Oryza sativa* seeds with increased concentrations of pristine ZnO NPs (0, 5, 10, 15, and 25 ppm) has been reported to encourage an increase in plant height and total chlorophyll contents (Mazhar et al., 2022). Previously, a study on the effects of different concentrations of ZnO NPs (0, 2, 4, 6, 8, 10, 100, 1,000, and 5,000 mg/L) compared to ionic ZnSO_4_, on *Phaseolus vulgaris* seed germination indicated that the 20 and 60 nm ZnO (100 mg/L) treatments could promote the hair growth on the primary roots in 5-day-old seedlings (Savassa et al., 2018). However, at 5,000 mg/L these nano-oxides and ZnSO_4_ were found to be toxic, hindering the development of roots. Another related study showed that nanopriming of *O. sativa* seeds with the 375 nm CS/TPP NPs (50 μg/mL) resulted in increased germination percentage and seedling vigour index in 7-day-old seedlings in normal growth conditions and under salt stress (Soni et al., 2023). It has been inferred that adsorption of chitosan NPs onto seeds could initiate the biosynthesis of auxin, which stimulates coleoptile elongation (Lee et al., 2022). Furthermore, Hameed et al. (2020) reported that treatment of *O. sativa* seeds with Zn NPs-stabilized chitosan-polyvinyl alcohol (0.16%) elicited the biosynthesis of proteins and upregulated the activities of protease and α-amylase enzymes in comparison with the unprimed seeds.

Curiously, limited studies have taken up the effects of metal oxide-integrated chitosan NPs in nanopriming technology. Considering the afore-mentioned facts, it was anticipated that nanopriming of *Z. mays* seeds using solutions containing CS/TPP NPs loaded with ZnO NPs or Zn^2+^ ions may combine the surface adherence attribute of chitosan with the essentiality of Zn^2+^ ions and the increased cellular penetration (increased surface of contact) of nanoscale metal oxides, to advance the physiological characteristics of *Z. mays* seedlings (Dimkpa and Bindraban, 2016; Mujtaba et al., 2020; Manuja et al., 2021).

#### 3.3.1 Effects on germination of non-stressed seeds

**FIGURE 9:**
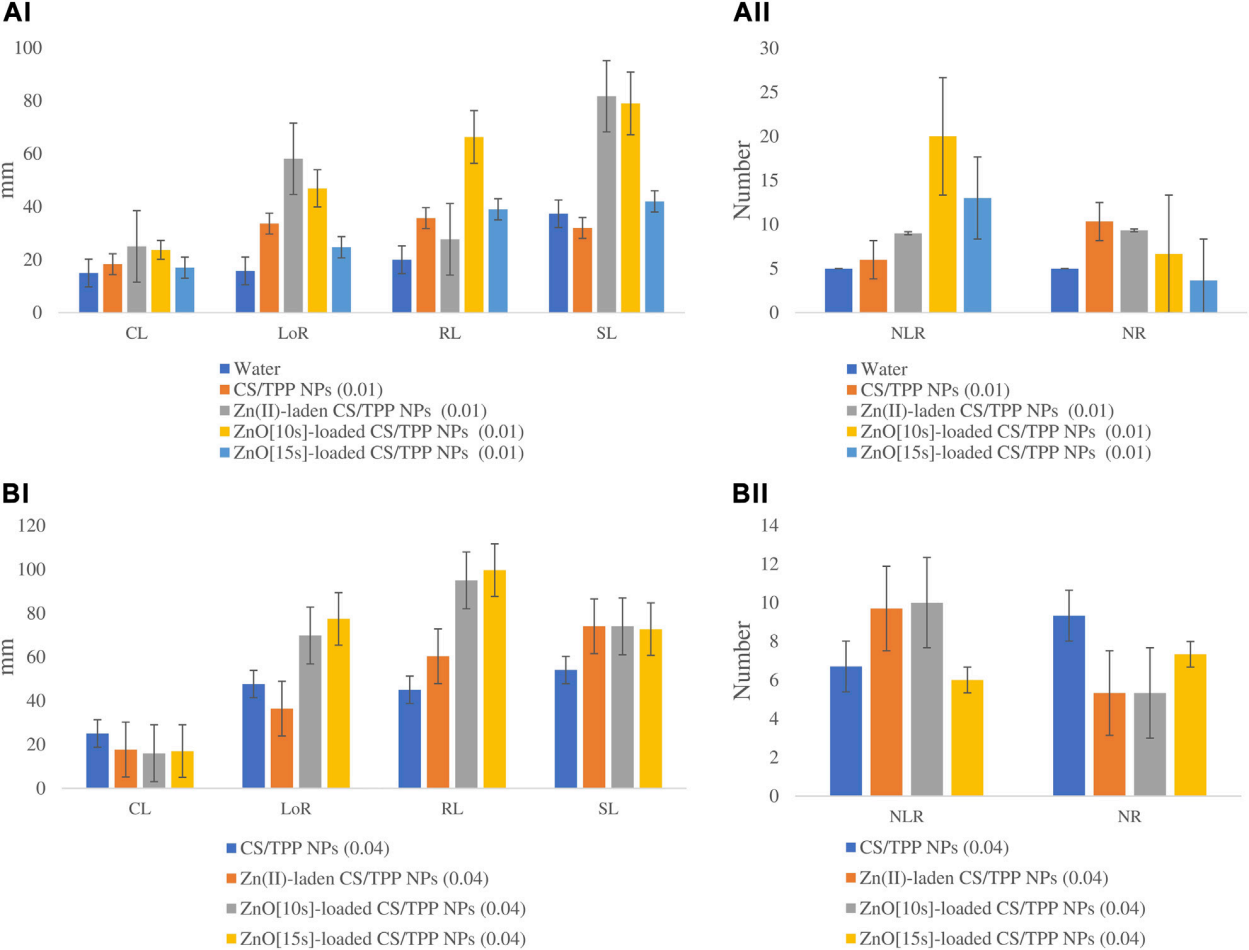
Effects of the prepared CS/TPP, Zn(II)-laden CS/TPP, and ZnO-loaded CS/TPP NPs at (A) 0.01% and (B) 0.04% on selected growth parameters [tissue length **(AI, BI)** and tissue number **(AII, BII)**] under salt stress (NaCl 600 mM). Means were recorded from replicates for each treatment and significantly different at *p ≤ 0.05*.

The seedlings from CS/TPP, Zn(II)-laden CS/TPP, ZnO [10s]-loaded CS/TPP, and ZnO [15s]-loaded CS/TPP nanopriming at 0.01% displayed significantly longer coleoptiles compared to the control set (Figure 9A). CS/TPP NPs treatment at a higher dose of 0.04% led to a maximum increase in mean CL 25.2 mm (Figure 9B). Seed priming with bulk chitosan and nano-engineered chitosan has been described for improved germination and seedling establishment (Ling et al., 2022; Samaneh Attaran Dowom et al., 2022). The mean coleoptile length for the control treatment (water) was recorded to be 15.0 mm. The highest length of roots was obtained from the ZnO [15s]-loaded CS/TPP and ZnO [10s]-loaded CS/TPP treatments at 0.04%. A study by Ozturk et al. (2006) noted that high Zn concentrations are required in highly metabolically active differentiating cells. Zn is highly mobile in germinating seeds and is needed in the actively growing parts of plants.

The mean LoR from the nanopriming agents ZnO [15s]-loaded CS/TPP and ZnO [10s]-loaded CS/TPP were recorded to be 77.4 and 69.8 mm, respectively. Maximum NLR 20.0 was found from the seeds treated with ZnO [10s]-loaded CS/TPP NPs at 0.01%. The control treatment showed the least average NLR 5.0. A report by Iglesias et al. (2019) showed that the 2.1 μm CS/TPP (10 μg/mL) could act as a biostimulant to achieve a 60% of increment in the NLR in *Arabidopsis thaliana* seedlings upon 48 h exposure compared to the untreated seedlings. This modulation of the root system architecture was attributed to the activation of the auxin signaling response. At 0.01% of our nanopriming agent, the highest average NR was obtained from the CS/TPP treatment (10.3), followed by Zn(II)-laden CS/TPP (9.3). Previously, Choudhary et al. (2019) reported that nanopriming maize seeds with the 387 nm Zn(II)-laden CS/TPP NPs (0.01%) could stimulate an increase in the NR in 9-day-old seedlings (6.0) compared to water as a control (5 6). This literature highlighted that the developed chitosan-based nanomaterial triggers higher activities of α-amylase and protease enzymes in seeds.

At an increased concentration of 0.04%, the highest NR was counted from the seeds treated with CS/TPP NPs (9.3). The ZnO [15s]-loaded CS/TPP and ZnO [10s]-loaded CS/TPP treatments at 0.04% also promoted maximum radicle length of 99.7 and 95.0 mm, respectively. The average shoot length was significantly higher from the Zn(II)-laden CS/TPP 0.01% (81.7 mm), ZnO [10s]-loaded CS/TPP 0.01% (79.0 mm), ZnO [10s]-loaded CS/TPP 0.04% (74.0 mm), and ZnO [15s]-loaded CS/TPP 0.04% (72.7 mm) compared to the control group with 42.4 mm. In terms of the growth parameters LoR and RL, the ZnO [15s]- and ZnO [10s]-loaded CS/TPP NPs at 0.04% achieved the best results. The overall growth traits presented in Table 4 showed that while the pristine CS/TPP NPs and ZnO [15s]-loaded CS/TPP NPs achieved superior promoting impact from 0.01% to 0.04%, the ZnO [10s]-loaded CS/TPP NPs would tend to be less effective at 0.04%. This observation suggested that at higher concentrations (>0.04%) of the ZnO [10s]-loaded CS/TPP NPs, a significant level of inhibitory effects could be observed on maize seed germination and plant growth (Yang et al., 2015). One may wonder if the toxicity of this nanocomposite could be from the physico-chemical interferences of the carrier-free nano-oxide with the germinating embryonic roots. *Z. mays* seed coat has been argued to provide protection from the 30 nm ZnO NPs entering seeds due to selective permeability, and in so doing mitigate their toxicity (Zhang et al., 2015). Lin and Xing, (2008) demonstrated that single particle of the 279 nm ZnO NPs could enter into the root cell of *Lolium perenne* and inhibit seedling growth. Therefore, the toxicity of the developed 257 nm ZnO [10s]-loaded CS/TPP NPs could be related to the free ZnO [10s]. Nonetheless, this postulation deserves further investigation as the Zn(II)-laden CS/TPP NPs priming solution also showed lower growth attributes at 0.04% compared to 0.01%.

**TABLE 4:** Growth traits for 10-day-old seedlings under non-stress conditions.

Treatment	CL	LoR	NLR	NR	RL	SL
Water	15.00^b^	15.80^c^	5.00^a^	5.00^b^	20.00^a^	37.30^b^
CS/TPP NPs (0.01%)	18.30^a^	33.60^b^	6.02^a^	10.33^a^	35.70^a^	32.00^b^
Zn(II)-laden CS/TPP NPs (0.01%)	25.00^a^	58.10^a^	9.00^a^	9.33^a^	27.70^a^	81.70^a^
ZnO [10s]-loaded CS/TPP NPs (0.01%)	23.70^a^	46.90^b^	20.00^a^	6.67^b^	66.30^a^	79.00^a^
ZnO [15s]-loaded CS/TPP NPs (0.01%)	17.01^a^	24.71^b^	13.00^a^	3.66^b^	39.00^a^	42.00^a^
CS/TPP NPs (0.04%)	25.00^a^	47.60^b^	6.70^a^	9.33^a^	45.00^a^	54.00^a^
Zn(II)-laden CS/TPP NPs (0.04%)	17.70^a^	36.40^a^	9.70^a^	5.33^b^	60.30^a^	74.00^a^
ZnO [10s]-loaded CS/TPP NPs (0.04%)	16.00^a^	69.80^a^	10.00^a^	5.33^b^	95.00^a^	74.00^a^
ZnO [15s]-CS/TPP NPs (0.04%)	16.99^a^	77.41^a^	6.00^a^	7.32^a^	99.70^a^	72.70^a^

Variables with different letters in the same column are significantly different at 5% probability level (*p ≤ 0.05*).

The coleoptile, radicle, shoot, and root dry mater productions were also significantly affected in the nanopriming systems compared to the control (Table 5). The seeds treated with only water (control) exhibited increased CDW (0.035 g) and SDW (0.052 g) as compared with the seeds exposed to nanoparticles. The second highest CDW and SDW were averaged to be 0.032 g for seeds treated with ZnO [10s]-loaded CS/TPP NPs 0.01%. A comparable mass (0.031 g) was also recorded using Zn(II)-laden CS/TPP NPs 0.04%. The highest mean RDW (0.015 g) and RoDW (0.033 g) were found from seeds primed with ZnO [10s]-loaded CS/TPP NPs 0.01% and Zn(II)-laden CS/TPP NPs 0.01%, respectively. Seed priming with Zn(II)-laden CS/TPP NPs 0.01%, ZnO [10s]-loaded CS/TPP NPs 0.01%, CS/TPP NPs (0.04%), and ZnO [15s]-loaded CS/TPP NPs (0.04%) showed a significant increase (>2-fold) in the dry weight of the roots in 10-day-old seedlings in comparison with the control.

**TABLE 5:** Effects of pristine CS/TPP NPs and Zn-loaded CS/TPP NPs on dry matter yield (g) for the seeds germinated under normal conditions.

Treatment	CDW	RDW	SDW	RoDW
Water	0.035^a^	0.009^c^	0.052^a^	0.011^c^
CS/TPP NPs (0.01%)	0.014^c^	0.006^d^	0.012^d^	0.018^b^
Zn(II)-laden CS/TPP NPs (0.01%)	0.027^b^	0.005^e^	0.024^c^	0.033^a^
ZnO [10s]-loaded CS/TPP NPs (0.01%)	0.032^a^	0.015^a^	0.032^b^	0.028^a^
ZnO [15s]-loaded CS/TPP NPs (0.01%)	0.018^c^	0.007^a^	0.008^c^	0.007^c^
CS/TPP NPs (0.04%)	0.018^c^	0.008^c^	0.020^c^	0.021^b^
Zn(II)-laden CS/TPP NPs (0.04%)	0.012^c^	0.011^b^	0.031^b^	0.012^c^
ZnO [10s]-loaded CS/TPP NPs (0.04%)	0.012^c^	0.010^b^	0.020^c^	0.018^b^
ZnO [15s]-loaded CS/TPP NPs (0.04%)	0.015^c^	0.008^c^	0.020^c^	0.025^b^

#### 3.3.2 Effects on germination of salt-stressed seeds

**FIGURE 10:**
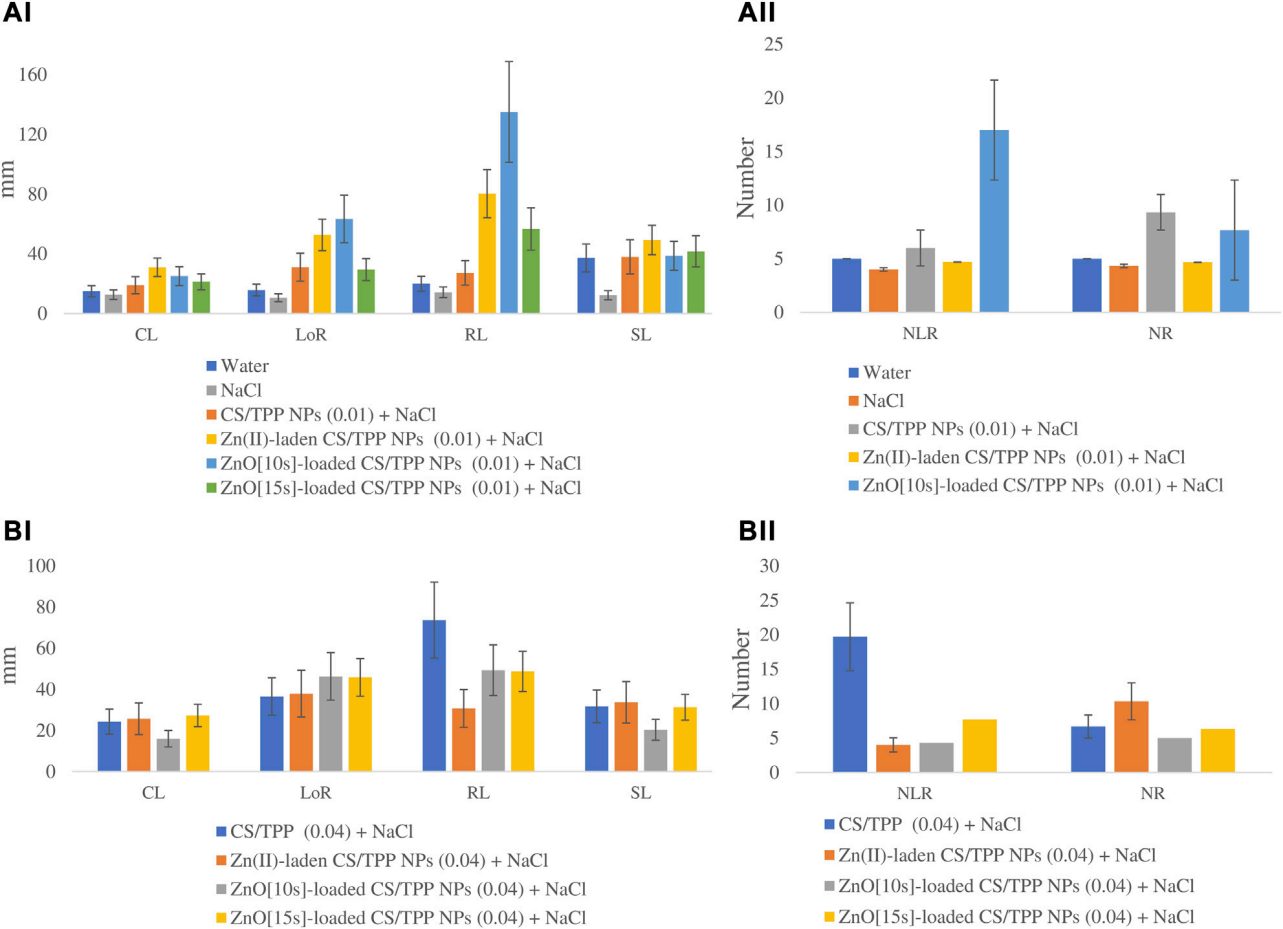
Effects of the prepared CS/TPP, Zn(II)-laden CS/TPP, and ZnO-loaded CS/TPP NPs at (A) 0.01% and (B) 0.04% on the growth parameters [tissue length **(AI, BI)** and tissue number **(AII, BII)**] under salt stress (NaCl 600 mM). Means were recorded from replicates for each treatment and significantly different at *p ≤ 0.05*.

The seeds primed with Zn(II)-laden CS/TPP NPs (0.01% and 0.04%), ZnO [10s]-loaded CS/TPP NPs 0.01%, ZnO [15s]-loaded CS/TPP NPs 0.04% and CS-TPP 0.04% under salinity stress had the longest mean CL 31.0, 25.7, 25.1, 27.3, 25, and 24.3 mm, respectively (Figure 10 (a and b), Table 6). The control group and seeds germinated under salinity conditions, with no addition of nanoparticles, displayed significantly lower average CL. The length of roots was recorded from the highest performed treatment to the least. The ZnO [10s]-loaded CS/TPP NPs 0.01% priming solution significantly increased the root length (>75%) of seedlings developing under salinity stress than the control group. The mean LoR of the seeds exposed to ZnO [10s]-loaded CS/TPP NPs 0.01%, Zn(II)-laden CS/TPP NPs 0.01%, ZnO [10s]-loaded CS/TPP NPs 0.04%, and ZnO [15s]-loaded CS/TPP NPs 0.04% were 63.4, 52.7, 46.3, and 45.8 mm, respectively. A considerable inhibition of root growth was observed with the NaCl set (LoR 13.6 mm), relative to the control group (LoR 15.8 mm). Salinity conditions reduce nutrient uptake and the rate of cell division at the root meristems, thereby impairing the seed germination and early seedling growth (Ondrasek et al., 2022). It is worth mentioning that knowledge of the stages of salt stress in *Z. mays* suggests that Na^+^ ions represent the main species causing toxicity rather than Cl^−^ (Wang et al., 2021). It is foreseen that the nanocomposites described in this study mitigate salinity stress by reducing the absorption of Na^+^ ions in the seeds and seedlings. These nanoparticles release Zn^2+^ ions (radius 0.074 nm) that can be easily absorbed by plant tissues than Na^+^ ions (radius 0.102 nm).

**TABLE 6:** Growth traits for 10-day-old seedlings under salt stress conditions.

Treatment	CL	LoR	NLR	NR	RL	SL
Water	15.00^b^	15.80^b^	5.00^a^	5.00^a^	20.00^c^	37.30^a^
NaCl	12.70^b^	10.60^b^	4.00^a^	4.33^a^	14.30^c^	12.30^b^
CS/TPP NPs (0.01%) + NaCl	19.00^b^	31.10^a^	6.00^a^	9.33^a^	27.30^c^	38.00^a^
Zn(II)-laden CS/TPP NPs (0.01%) + NaCl	31.00^a^	52.70^a^	4.70^a^	4.67^a^	80.30^b^	49.30^a^
ZnO [10s]-loaded CS/TPP NPs (0.01%) + NaCl	25.10^a^	63.40^a^	17.00^a^	7.67^a^	135.00^a^	38.70^a^
ZnO [15s]-loaded CS/TPP NPs (0.01%) + NaCl	21.30^a^	29.50^a^	6.30^a^	7.00^a^	56.71^b^	41.7.0^a^
CS/TPP NPs (0.04%) + NaCl	24.30^a^	36.50^a^	19.70^a^	6.67^a^	73.70^b^	31.70^a^
Zn(II)-laden CS/TPP NPs (0.04%) + NaCl	25.70^a^	37.90^a^	4.00^a^	10.33^a^	30.70^c^	33.70^a^
ZnO [10s]-loaded CS/TPP NPs (0.04%) + NaCl	16.00^b^	46.30^a^	4.30^a^	5.00^a^	49.30^b^	20.30^b^
ZnO [15s]-loaded CS/TPP NPs (0.04%) + NaCl	27.30^b^	45.80^a^	7.70^a^	6.33^a^	48.70^b^	31.30^a^

Regarding the number of lateral roots, the CS/TPP NPs 0.04% and ZnO [10s]-loaded CS/TPP NPs 0.01% treatments resulted in better growth (NLR 19.7 and 17.0, respectively) compared to other groups. The highest average number of roots was recorded from the seed priming with Zn(II)-laden CS/TPP NPs 0.04% (10.3), followed by CS/TPP NPs 0.01% (9.3). The root growth inhibition was also evidenced by the NaCl treatment (NR 4.3). Furthermore, the mean radicle length from seeds primed with ZnO [10s]-loaded CS/TPP NPs 0.01%, Zn(II)-laden CS/TPP NPs 0.01%, CS/TPP NPs 0.04%, and ZnO [10s]-loaded CS/TPP NPs 0.04% was 6.7-fold, 4.0-fold, 3.7-fold, and 2.8-fold greater than the control. When analyzing the shoot length, Zn(II)-laden CS/TPP NPs 0.01%, ZnO [15s]-loaded CS/TPP NPs 0.01%, and CS/TPP NPs 0.01% treatments had the best variables (SL 49.3, 41.7 and 38.0 mm, respectively). Seed priming with CS/TPP NPs, ZnO [10s]-loaded CS/TPP NPs, and Zn(II)-laden CS/TPP NPs at 0.01% influenced the early development of the salt-stressed seeds, mainly by increasing CL, LoR, NLR, and NR compared to the control. These chitosan-based nanostructures are anticipated to counteract the inhibitory effects of salinity conditions by triggering the production of intracellular messengers, H_2_O_2_, nitric oxide (NO), and Ca^2+^ (Hu et al., 2016; Hidangmayum et al., 2019), which induce physiological responses. In addition, the antioxidant properties of chitosan could alleviate the oxidative stress caused by salinity (Jabeen and Ahmad, 2013). In agreement with the findings presented here, Abdel-Aziz (2019) reported that priming of *Vicia faba* seed with CS/methacrylic acid NPs 0.05% boosted the activities of polyphenol oxidase, peroxidase, ascorbate peroxidase, and catalase under salt stress. The scavenging action of chitosan against ROS is attributed to the reactivity of its–OH and–NH_2_ groups.

## 4 Conclusion

This work aimed to develop nanofertilizer-based seed priming agents using a green, facile, and cost-effective strategy. ZnO nanoparticles of different sizes, as nutrients, were prepared from aqueous extracts of South Africa’s indigenous plant *H. odoratissimum* at different concentrations without the need for calcination. XRD was used to verify their crystalline structure. TEM and XPS analysis confirmed the successful integration of these nano-zinc oxides and zinc ionic species within TPP-crosslinked CS nanoparticles. The colloidal suspensions of the ZnO-loaded CS/TPP nanocomposites exhibit a slow release of Zn(II) ions in water over 02 weeks. Maize seed priming with solutions of these combinative ZnO-chitosan nanoproducts promotes the radicle, root, and shoot growth. On the other hand, low-concentration (0.01%) CS/TPP NPs and Zn(II)-laden CS/TPP NPs improve the early development of the salt-stressed seeds. These results demonstrate the effectiveness of the developed nanofertilizers in increasing the germination rate of maize seeds, even under salinity conditions.

## Data Availability

The original contributions presented in the study are included in the article/Supplementary material, further inquiries can be directed to the corresponding authors.
